# MPTP Impairs Dopamine D1 Receptor-Mediated Survival of Newborn Neurons in Ventral Hippocampus to Cause Depressive-Like Behaviors in Adult Mice

**DOI:** 10.3389/fnmol.2016.00101

**Published:** 2016-10-13

**Authors:** Tingting Zhang, Juan Hong, Tingting Di, Ling Chen

**Affiliations:** ^1^State Key Lab of Reproductive Medicine, Nanjing Medical UniversityNanjing, China; ^2^Department of Physiology, Nanjing Medical UniversityNanjing, China

**Keywords:** Parkinson’s disease (PD), dopaminergic receptor (DR), depression, neurogenesis, hippocampal dentate gyrus (DG)

## Abstract

Parkinson’s disease (PD) is characterized by motor symptoms with depression. We evaluated the influence of dopaminergic depletion on hippocampal neurogenesis process to explore mechanisms of depression production. Five consecutive days of 1-Methyl-4-phenyl-1,2,3,6-tetrahydropyridine (MPTP) injection in mice (MPTP-mice) reduced dopaminergic fibers in hippocampal dentate gyrus (DG). MPTP-mice exhibited depressive-like behaviors later for 2–3 weeks. BrdU was injected 4 h after last-injection of MPTP. BrdU-positive (BrdU^+^) cells in dorsal (d-DG) and ventral (v-DG) DG were examined on day 1 (D1), 7 (D7), 14 (D14) and 21 (D21) after BrdU injection. Fewer D7-, D14- and D21-BrdU^+^ cells or BrdU^+^/NeuN^+^ cells, but not D1-BrdU^+^ cells, were found in v-DG of MPTP-mice than in controls. However, the number of BrdU^+^ cells in d-DG did not differ between the both. Loss of doublecortin-positive (DCX^+^) cells was observed in v-DG of MPTP-mice. Protein kinase A (PKA) and Ca^2+^/cAMP-response element binding protein (CREB) phosphorylation were reduced in v-DG of MPTP-mice, which were reversed by D1-like receptor (D1R) agonist SKF38393, but not D2R agonist quinpirole. The treatment of MPTP-mice with SKF38393 on days 2–7 after BrdU-injection reduced the loss of D7- and D21-BrdU^+^ cells in v-DG and improved the depressive-like behaviors; these changes were sensitive to PKA inhibitor H89. Moreover, the v-DG injection of SKF38393 in MPTP-mice could reduce the loss of D21-BrdU^+^ cells and relieve the depressive-like behaviors. In control mice, the blockade of D1R by SCH23390 caused the reduction of D21-BrdU^+^ cells in v-DG and the depressive-like behaviors. Our results indicate that MPTP-reduced dopaminergic depletion impairs the D1R-mediated early survival of newborn neurons in v-DG, producing depressive-like behaviors.

## Introduction

Parkinson’s disease (PD) is a neurodegenerative disorder characterized by motor symptoms and a progressive loss of dopaminergic neurons (Rodriguez-Oroz et al., 2009). Major depression is present in approximately 30–50% of PD patients (Bower et al., 2010; Nègre-Pagès et al., 2010). However, the underlying mechanisms remain unclear.

The hippocampal volume is smaller in patients with depression than in normal subjects of the same-age (Videbech and Ravnkilde, 2015). Electroconvulsive therapy in refractory depression can increase hippocampal volume. Growing evidence suggests that neurogenesis continues throughout adulthood within the hippocampal dentate gyrus (DG; Toni et al., 2008). The newly generated neurons can integrate into hippocampal circuitry, thereby maintaining a functional structure, which is required for mood control and antidepressant efficacy (Petrik et al., 2012). The deficits in adult neurogenesis in the postmortem brains of PD patients have recently attracted much attention. For example, Höglinger et al. (2004) have reported the deficits in the hippocampal neurogenesis of PD patients. The hippocampal cell proliferation capacity is decreased in PD patients (Guiard et al., 2009). A reduction in neuronal precursor cells has been observed in the subgranular zone (SGZ) of patients with PD (Borta and Höglinger, 2007).

The hippocampus and the midbrain dopaminergic neurons of the ventral tegmental area (VTA) form a functional loop (Lisman and Grace, 2005). Dopaminergic fibers, originating mainly from VTA (Gasbarri et al., 1997), directly contact the newborn cells in DG, but are sparse in the granule cell layer or adjacent hilus (Höglinger et al., 2004). Neuronal precursor cells in the hippocampal DG of adult mammals express all dopaminergic receptors (DRs), which receive dopaminergic afferents (Takamura et al., 2014). Dopamine (DA) has been reported to play an important role in the regulation of endogenous neurogenesis in the adult mammalian brain. The activation of D1-like receptors (D1R) promotes the survival of newborn cells in the adult hippocampus (Takamura et al., 2014). Winner et al. (2009) have observed that a D2-like receptor (D2R) agonist in DA-depleted rats increases the proliferation of neural precursor cells in the subventricular zone (SVZ) but not in SGZ. A large body of evidence has established that the DA depletion in rodents decreases the cell proliferation and survival of neuronal precursor cells in DG (Höglinger et al., 2004; Khaindrava et al., 2011). However, other data have revealed that the DA depletion increases or has no effect on cell proliferation (Oizumi et al., 2008; Park and Enikolopov, 2010).

In rodents and primates, the changes in afferent and efferent connectivity along the longitudinal axis of the hippocampus suggest distinct functions of the dorsal and ventral hippocampus. The dorsal DG (d-DG) receives projections arising in the lateral and caudomedial portion of entorhinal cortex, whereas the ventral DG (v-DG) receives inputs from the rostromedial region of the entorhinal cortex (Dolorfo and Amaral, 1998). The innervation density of dopaminergic plexuses is very high in the ventral hippocampus, but is low in the dorsal portion (Bjarkam et al., 2003). 1-Methyl-4-phenyl-1,2,3,6-tetrahydropyridine (MPTP) is commonly used to prepare an animal model of PD as it selectively induces cell death of dopaminergic neurons (Schober, 2004). The MPTP lesion can be used for behavioral studies of affective disorders in C57BL/6 mice (Gorton et al., 2010). To investigate whether the DA depletion in PD brain impairs the hippocampal neurogenesis to cause the depression, we in this study examined the affective behaviors and the neurogenesis process of d-DG and v-DG (including the proliferation of stem cells, the differentiation of progenitor cells and the survival of newborn neurons) in mice treated with MPTP (MPTP-mice), and explored the correlation between hippocampal neurogenesis deficits and depressive-like behaviors. The present study provides evidence that MPTP-reduced dopaminergic afferents impairs the D1R-mediated early survival of newly generated neurons in v-DG, which may be responsible for the production of depressive-like behaviors.

## Materials and Methods

### Mice

The use of animals was approved by Institutional Animal Care and Use Committee of Nanjing Medical University and was performed in accordance with the experimental animal guidelines of Laboratory Animal Research Institute. Eight-week-old male C57BL/6 mice (24–26 g; Oriental Bio Service Inc., Nanjing, China) were used at the beginning of the experiment. The mice were maintained under constant environmental conditions (temperature 23 ± 2°C, humidity 55 ± 5% and 12:12 h light/dark cycle) in Animal Research Center of Nanjing Medical University with free access to food and water.

### Drug Administration

The mice received an intraperitoneal (i.p.) injection of MPTP (25 mg/kg, measured as free base; Sigma-Aldrich, St. Louis, MO, USA) once a day for five consecutive days (Crocker et al., 2003). BrdU (Sigma-Aldrich) was dissolved freshly in 0.9% saline to make 10 mg/ml solution just before injection. Mice were given three injections of BrdU (50 mg/kg, i.p.) at intervals of 6 h. SKF38393 (Tocris, UK), SCH23390 and quinpirole (Sigma-Aldrich) were dissolved in sterile saline; L-sulpiride and H89 (Sigma-Aldrich) were dissolved in 1.0% DMSO (Sutton and Caron, 2015). The mice were treated daily with the injection (i.p.) of SKF38393 (10 mg/kg), H89 (1 mg/kg; Seyedi et al., 2014), quinpirole (2 mg/kg), SCH23390 (0.5 mg/kg) or L-sulpiride (15 mg/kg). For the v-DG injection of drugs, the mice were anesthetized with chloral hydrate (400 mg/kg, i.p.) and placed in a stereotaxic instrument (Stoelting, Wood Dale, IL, USA). The scalp was incised and a small hole (2 mm diameter) was drilled in the skull using a dental drill. Guide cannulas (26-Gauge, Plastics One, Roanoke, VA, USA) were implanted into the bilateral v-DG (2.3 mm posterior, 1.3 mm lateral and 2.0 mm ventral to Bregma; Zhou et al., 2011b). On day 2 after surgery, the dummy cannulas were removed from the guide cannula, and then replaced by infusion cannulas (30 Gauge). The infusion cannula was connected by polyethylene tubing (PE 10; Becton Dickinson, Sparks, MD, USA) with a stepper-motorized micro-syringe (Stoelting, Wood Dale, IL, USA). SKF38393 (4.8 nmol), SCH23390 (0.6 nmol) and H89 (0.8 nmol) were diluted with ACSF and infused daily in a volume of 0.2 μl/side (Lai et al., 2008; Nasehi et al., 2011). After 2% Evans-blue (2.5 μl) was injected, the mice were killed by an overdose of chloral hydrate, and coronal sections (100 μm) were cut using a cryostat to validate the injection-site. Control mice were given an equal volume of vehicle.

### Behavioral Examination

A single cohort of animals was used for the following test sequence: open-field test (OFT) → Forced swim test (FST) → Tail suspension test (TST; Zhou et al., 2014). All behavioral data were captured by a video-monitor and analyzed using TopScan Lite 2.0 (Clever Sys., Reston, VA, USA).

OFT was performed in a cuboid Plexiglass box (60 cm × 60 cm × 40 cm). Total distance traveled (mm/6 min) was recorded.

FST was performed in a glass cylinder (300 mm high, 280 mm in diameter) that was filled with water (25 ± 1°C) to a height of 20 cm. Total immobility time during a 6 min test was scored. Mice were considered to be immobile when they stopped struggling and moved only to remain floating in the water, while keeping their heads above water.

TST was performed by using adhesive tape to attach the tail to a rod that was 60 cm above the floor. Trials were conducted for a period of 6 min, during which the immobility time was recorded.

Morris water maze task (MWM) was performed in a black-colored plastic pool (diameter = 120 cm) at 20 ± 1°C. A cylindrical platform (diameter = 7 cm) was placed 0.5 cm below the surface of water. Each mouse was randomly released from four different quadrants and allowed to swim for 90 s. Four trials were conducted each day with an intertrial interval of 30 min. Average swimming speed (m/s) and latency (s) to reach the platform were scored for all trials. If a mouse could not reach the platform within 90 s, the experimenter gently assisted the mouse onto the platform and allowed it to remain there for 15 s.

### Immuno-Staining and Quantification

#### BrdU Immuno-Staining

Mice were anesthetized with chloral hydrate (400 mg/kg, i.p.) and perfused transcardially with 4% paraformaldehyde on day 1 (D1), day 7 (D7) , day 14 (D14) and day 21 (D21) after the last-injection of BrdU. Coronal hippocampal slices (40 μm) were cut using a vibrating microtome (Microslicer DTK 1500; Dousaka EM Co, Japan). The d-DG (AP: −0.94 to −2.30) and the v-DG (AP: −2.46 to AP: −3.80) were each harvested on the basis of the coordinates of the Paxinos and Franklin atlas of the mouse brain (O’Leary et al., 2012). The free-floating sections were treated with 3% normal goat serum, and then incubated with mouse anti-BrdU antibody (1:1000, Millipore, Billerica, MA, USA) at 4°C overnight. The sections were incubated in biotin-labeled goat anti-mouse IgG antibody (1:500, Bioworld Technology, Inc., St. Louis Park, MN, USA) for 2 h. Immunoreactivities were visualized using an avidin-biotin horseradish peroxidase complex (Vector Laboratories, Inc., Burlingame, CA, USA). BrdU-positive (BrdU^+^) cells in SGZ and granule cell layer of every 5th section (200 μm apart) were counted using a conventional light microscope (DP70, Olympus Optical, Tokyo, Japan). The number of BrdU^+^ cells per section was multiplied by five to obtain the total number per DG (Sha et al., 2015).

#### BrdU and NeuN or GFAP Double Immuno-Staining

The sections were incubated with rat anti-BrdU antibody (1:200, Abcam, Cambridge, UK), which was detected using CY3-labeled anti-rat IgG antibody (1:200, Millipore) and mouse anti-neuronal nuclei (NeuN) antibody (1:500, Millipore), which was detected using fluorescein-labeled anti-mouse antibody (1:50, Millipore) or mouse anti-GFAP antibody (1:200, Millipore), which was detected using a FITC-labeled anti-mouse antibody (1:50, Millipore). BrdU^+^/NeuN^+^ or GFAP^+^ cells were observed using a confocal laser-scanning microscope (Leica, Heidelberg, Germany). The number of BrdU^+^/NeuN^+^ or GFAP^+^ cells per section (200 μm apart) was multiplied by 5 to obtain the total number.

#### Doublecortin (DCX) or Tyrosine Hydroxylase (TH) Immuno-Staining

The free-floating sections (40 μm) were incubated with goat anti-DCX antibody (1:500, Santa Cruz, CA, USA) or chicken anti-TH (1:1000, Abcam) at 4°C overnight, and then in biotin-labeled rabbit anti-goat IgG antibody (1:500, Bioworld) or goat anti-chicken IgG antibody (1:500, Santa Cruz) for 2 h at room temperature. Density of doublecortin positive (DCX^+^) cells was expressed as the number per mm length along SGZ (Sha et al., 2015). Tyrosine hydroxylase positive (TH^+^) cells were counted using a stereological system, which consisted of a light microscope with a CCD camera (DP70), a motorized specimen stage for automatic sampling and a computer running Microbrightfield Stereo Investigator software (Microbrightfield, Williston, VT, USA; Hong et al., 2015).

### Western Blot Analysis

After the dorsal and ventral hippocampal slices were harvested, the DG regions were micro-dissected and stored at −80°C until assayed. Protein was extracted and the concentration of protein was determined using a bicinchoninic acid (BCA) protein assay kit (Pierce, IL, USA). Protein (40 μg) was separated by 10% acrylamide denaturing gels (SDS-PAGE) and transferred to membranes. The membranes were incubated with rabbit anti-protein kinase A (PKA) phosphorylation (1:1000, Millipore) and rabbit anti-cyclic AMP-response element binding protein (CREB) phosphorylation (1:1000, Santa Cruz, CA, USA). Then, the membranes were incubated with horseradish peroxidase-labeled goat anti-rabbit antibody (1:5000, Santa Cruz, CA, USA), and developed using an enhanced chemiluminescence detection kit (Millipore). After visualization, the blots were stripped by incubation in stripping buffer for 15 min, and then incubated with rabbit anti-PKA antibody (1:1000, Millipore) and rabbit anti-CREB antibody (1:1000, Santa Cruz, CA, USA). An internal control was performed using mouse anti-β-actin antibody (1:2000, Cell Signaling, Danvers, MA, USA). Western blot bands were scanned and analyzed with the ImageJ analysis software package (NIH).

### Reverse Transcription-Polymerase Chain Reaction (RT-PCR)

Total RNA of v-DG and d-DG was isolated using TRIzol reagent (Invitrogen, Camarillo, CA, USA) and reverse-transcribed into cDNA using a PrimeScript RT reagent kit (Takara, China) for quantitative PCR (ABI Step One Plus, Foster City, CA, USA) in the presence of a fluorescent dye (SYBR Green I; Takara). The relative expression of genes was determined using the 2^−ΔΔct^ method with normalization to the GAPDH expression. The primer sequences of *D1R* and *D2R* mRNA were designed as described in a previous publication (Kim et al., 2010).

### Statistical Analysis

The group data were expressed as the means ± standard error (SEM). All statistical analyses were performed using SPSS software, version 16.0 (SPSS Inc., Chicago, IL, USA). Differences among means were analyzed using the Student’s *t*-tests or one/two-factor analysis of variance (ANOVA) or repeated-measures ANOVA, followed by a *post hoc* Bonferroni test. Differences of *p* < 0.05 were considered statistically significant.

## Results

### MPTP Causes Depression-Like Behaviors in Mice

**Figure 1:**
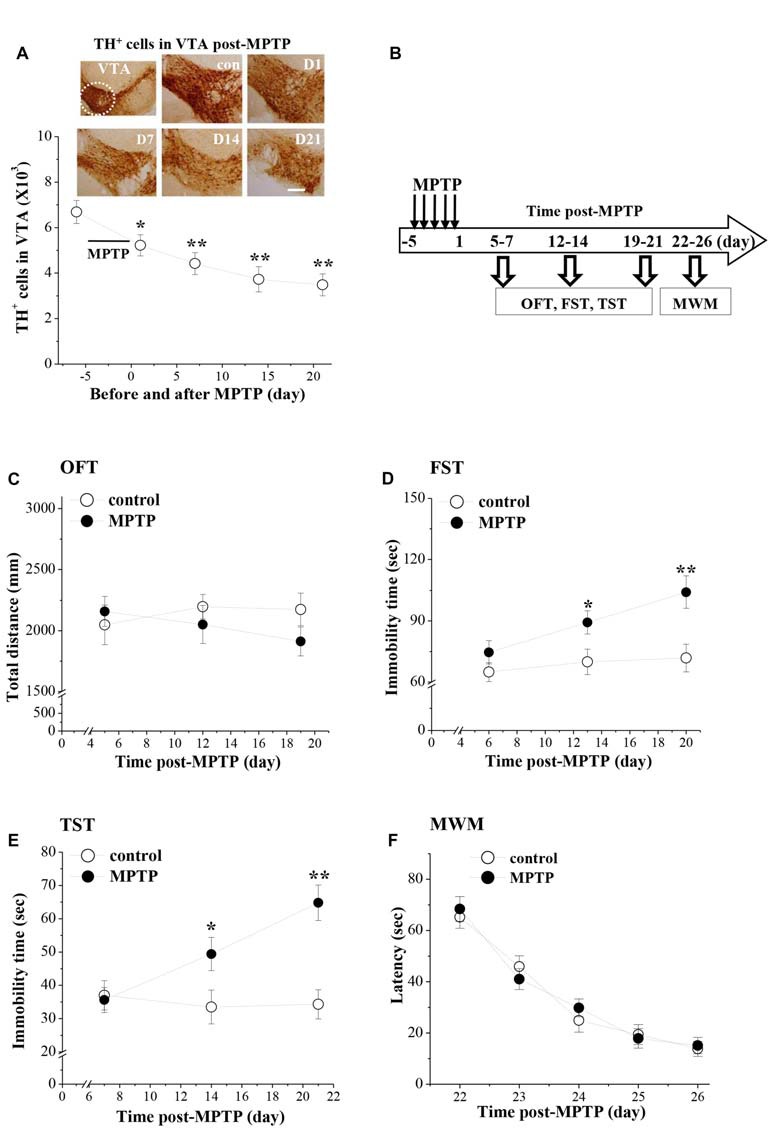
**Influence of 1-Methyl-4-phenyl-1,2,3,6-tetrahydropyridine (MPTP)-induced dopamine (DA) depletion on affective behaviors in mice. (A)** Representative photomicrographs of tyrosine hydroxylase (TH) immuno-staining in ventral tegmental area (VTA; a circular area is indicated by white line). Scale bars = 40 μm. Stereological counts of TH^+^ cells throughout VTA on day 1 (D1), 7 (D7), 14 (D14) and 21 (D21) after the last-injection of MPTP (post-MPTP). *p < 0.05 and **p < 0.01 vs. control mice (one-way ANOVA). **(B)** Time chart of the experimental procedure. Horizontal open arrow indicates the time post-MPTP (day). Vertical open arrows indicate the time of open-field test (OFT), forced swim test (FST) and tail suspension test (TST) and Morris water maze task (MWM). **(C)** Each point represents the total distance traveled within 6 min in OFT on day 5, 12 and 19 post-MPTP, respectively. **(D,E)** The points indicate the immobility time in FST and TST on days 6–7, days 13–14 and days 20–21 post-MPTP, respectively. *p < 0.05 and **p < 0.01 vs. control mice (two-way ANOVA). **(F)** Each point represents the mean latencies (sec) to reach the hidden platforms on days 22–26 post-MPTP.

MPTP injection had a marked effect on the dopaminergic neurons in VTA (*F*_(4,49)_ = 6.767, *p* < 0.001; Figure 1A). In comparison with the control values, the number of TH^+^ cells on day 1 (D1, *p* < 0.05), day 7 (D7, *p* < 0.01), day 14 (D14, *p* < 0.01) and day 21 (D21, *p* < 0.01) after the last-injection of MPTP (post-MPTP) was significantly reduced.

The affective behaviors were examined on days 5–7, days 12–14 and days 19–21 post-MPTP (Figure 1B). MPTP-mice had a tendency to travel a shorter total distance in the OFT (Figure 1C), but the group comparison with control mice failed to reach statistical difference (*F*_(1,54)_ = 0.825, *p* = 0.368). Depression-like behaviors were examined by FST and TST. There was a main effect of MPTP-injection for the immobility time in FST (*F*_(1,54)_ = 16.130, *p* < 0.001; Figure 1D) and TST (*F*_(1,54)_ = 15.360, *p* < 0.001; Figure 1E). Notably, MPTP-mice showed a progressive prolongation of immobility time in FST and TST from day 13–14 (FST: *p* < 0.05; TST: *p* < 0.05) to day 20–21 (FST: *p* < 0.01; TST: *p* < 0.01) post-MPTP.

The spatial memory was further examined using the place learning of the MWM on days 22–26 post-MPTP. Repeated-measures ANOVA revealed that the escape latency of hidden platform progressively decreased with training days in all groups (*F*_(4,72)_ = 59.308, *p* < 0.001; Figure 1F), which was not affected by the MPTP-injection (*F*_(1,18)_ = 0.049, *p* = 0.828).

### MPTP Impairs the Survival of Newborn Neurons in Ventral DG

**Figure 2:**
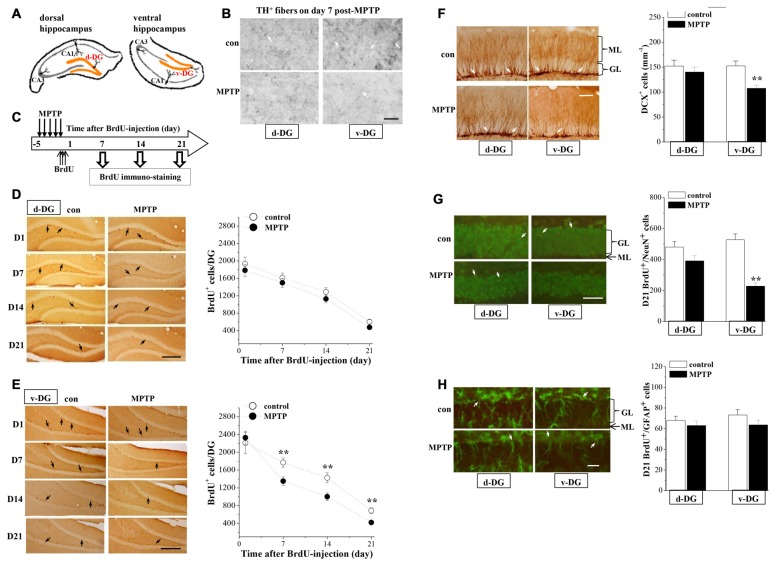
**Influence of MPTP-induced DA depletion on neurogenesis in dentate gyrus (DG). (A)** Schematic diagram of dorsal DG (d-DG) and ventral DG (v-DG; orange area). **(B)** Representative pictures of TH^+^ fibers (white arrows) in d-DG and v-DG on day 7 post-MPTP. Scale bars = 50 μm. **(C)** Time chart of the experimental procedure. Horizontal open arrow indicates the time after BrdU-injection (day). Vertical open arrows indicate the time of BrdU immuno-staining. **(D,E)** Representative images of BrdU-positive (BrdU^+^) cells (black arrows) in d-DG **(D)** and v-DG **(E)**. Scale bar = 100 μm. The points represent the mean number of D1-, D7-, D14- and D21-BrdU^+^ cells in d-DG and v-DG. **p < 0.01 vs. control mice (two-way ANOVA). **(F)** Bar graph shows the mean number of DCX-positive (DCX^+^) cells in d-DG and v-DG on day 7 post-MPTP. **p < 0.01 vs. control mice (Student’s *t*-tests). Representative images of DCX^+^ cells (white arrows). ML: molecular layer; GL: granular cell layer. Scale bar = 50 μm. **(G,H)** Bar graph shows the mean number of D21-BrdU^+^/NeuN^+^ cells and D21-BrdU^+^/GFAP^+^ cells in d-DG and v-DG. **p < 0.01 vs. control mice. Representative images of BrdU^+^/NeuN^+^ cells **(G)** and BrdU^+^/GFAP^+^ **(H)** cells (white arrows). Neuronal nuclei (NeuN) and glial fibrillary acidic protein (GFAP) are shown in green and BrdU is shown in red, newborn cells are shown in yellow. Scale bars = 50 μm.

To investigate the mechanisms underlying the MPTP-induced depression-like behaviors, we examined the dopaminergic innervations and the hippocampal neurogenesis in d-DG and v-DG (Figure 2A). In control mice, the density of TH positive (TH^+^) fibers in v-DG was higher than that in d-DG (Figure 2B). In comparison with those of controls, the TH^+^ fibers in v-DG were clearly decreased on day 7 post-MPTP. The BrdU was injected starting from 4 h post-MPTP. BrdU^+^ cells were examined on day 1 (D1), day 7 (D7), day 14 (D14) and day 21 (D21) after the BrdU-injection (Figure 2C). The number of BrdU^+^ cells (D1→D7→D14→D21) in either d-DG (*F*_(3,72)_ = 57.836, *p* < 0.001; Figure 2D) or v-DG (*F*_(3,72)_ = 64.856, *p* < 0.001; Figure 2E) were decreased progressively in all groups. MPTP injection markedly reduced the number of BrdU^+^ cells in v-DG (*F*_(1,72)_ = 7.800, *p* = 0.007), but not in d-DG (*F*_(1,72)_ = 3.497, *p* = 0.066). In comparison with controls, the MPTP-mice had significantly fewer D7-BrdU^+^ cells (*p* < 0.01), D14-BrdU^+^ cells (*p* < 0.01) and D21-BrdU^+^ cells (*p* < 0.01), but not D1-BrdU^+^ cells (*p* > 0.05), in v-DG. However, the numbers of D1-BrdU^+^ cells (*p* > 0.05), D7-BrdU^+^ cells (*p* > 0.05), D14-BrdU^+^ cells (*p* > 0.05) and D21-BrdU^+^ cells (*p* > 0.05) in d-DG had no significant difference between MPTP-mice and control mice.

Doublecortin (DCX), a microtubule-associated protein, is specifically expressed in immature neuroblasts and progenitor cells (Francis et al., 1999). To evaluate the survival of young newborn neurons, we examined the number of DCX^+^ cells in the hippocampal DG on day 7 post-MPTP. The results showed that the number of DCX^+^ cells was significantly reduced in v-DG (*p* < 0.01, *n* = 10; Figure 2F), but not in d-DG (*p* > 0.05, *n* = 10), compared with those in control mice.

To examine the differentiation of precursor cells, we further examined the number and relative proportions of D21-BrdU^+^ cells expressing NeuN, a mature neuron marker, or glial fibrillary acidic protein (GFAP), a glial marker. The number of D21-BrdU^+^/NeuN^+^ cells in v-DG of MPTP-mice was less than that in control mice (*p* < 0.01, *n* = 10; Figure 2G), whereas there was no difference in d-DG between the both (*p* > 0.05, *n* = 10). In contrast, the number of D21-BrdU^+^/GFAP^+^ cells in v-DG (*p* > 0.05, *n* = 10; Figure 2H) or d-DG (*p* > 0.05, *n* = 10) did not differ between MPTP-mice and control mice. In v-DG of MPTP-mice, the ratio of BrdU^+^/NeuN^+^ cells to total D21-BrdU^+^ cells (53.79 ± 5.02%) was lower than that in control mice (76.60 ± 5.48%, *p* < 0.01, *n* = 10), whereas the ratio of BrdU^+^/GFAP^+^ cells (15.06 ± 1.09%) was higher than the ratio in control mice (10.54 ± 0.74%, *p* < 0.01, *n* = 10).

### MPTP Reduces D1R-Activated PKA-CREB Signaling

**Figure 3:**
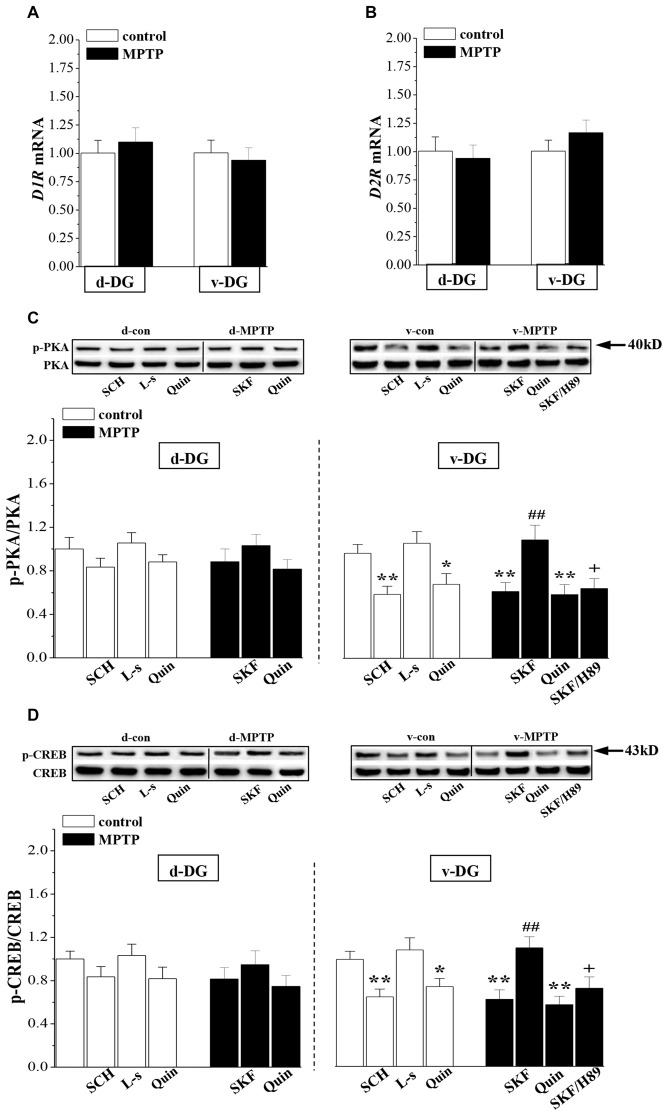
**Influence of MPTP-induced DA depletion on phosphorylation of PKA and CREB. (A,B)** Levels of D1-like receptors (*D1R*) and *D2R* mRNA in d-DG and v-DG on day 7 post-MPTP. **(C,D)** Bar graphs show the mean levels of PKA and CREB phosphorylation in d-DG and v-DG of MPTP-mice untreated or treated with SKF38393 (SKF, 10 mg/kg, i.p.), H89 (1 mg/kg, i.p.) or quinpirole (Quin, 2 mg/kg, i.p.) for 2 days and control mice untreated or treated with SCH23390 (SCH, 0.5 mg/kg, i.p.) or L-sulpiride (L-s, 15 mg/kg. i.p.) for 2 days. *p < 0.05 and **p < 0.01 vs. control mice; ^##^*p* < 0.01 vs. MPTP-mice; ^+^*p* < 0.05 vs. MPTP-mice treated with SKF.

The RT-PCR analysis showed that the levels of *D1R* (v-DG: *p* > 0.05; d-DG: *p* > 0.05, *n* = 8; Figure 3A) and *D2R* mRNA (v-DG: *p* > 0.05; d-DG: *p* > 0.05, *n* = 8; Figure 3B) were not altered on day 7 post-MPTP. Compared with the control levels, the phosphorylation of PKA (p-PKA: *p* < 0.01; Figure 3C) or CREB (p-CREB: *p* < 0.01, *n* = 8; Figure 3D) in v-DG was reduced on day 7 post-MPTP, which was reversed by the administration of D1R agonist SKF38393 for 2 days (*p* < 0.01, *n* = 8). The PKA inhibitor H89 could block the D1R-reversed CREB phosphorylation in v-DG of MPTP-mice (*p* < 0.05, *n* = 8). The administration of D1R antagonist SCH23390 for 2 days to control mice decreased the phosphorylation of PKA (*p* < 0.01) and CREB (*p* < 0.01, *n* = 8) in v-DG. Consistently with the results of a report by Zhao et al. (2013), the activation of D2R by quinpirole led to a decline in the phosphorylation of PKA (*p* < 0.05) and CREB (*p* < 0.05, *n* = 8) in v-DG of control mice, whereas the D2R antagonist L-sulpiride had no effects (*p* > 0.05, *n* = 8). The treatment of MPTP-mice with quinpirole showed a tendency to decrease the phosphorylation of PKA and CREB in v-DG, but the difference had no statistical significance (*p* > 0.05, *n* = 8). In contrast, MPTP-mice did not show the changes in the levels of the PKA and CREB phosphorylation (*p* > 0.05, *n* = 8) in d-DG compared with those of controls, which was not affected by SKF38393 (*p* > 0.05, *n* = 8) or quinpirole (*p* > 0.05, *n* = 8). Furthermore, the phosphorylation of PKA or CREB in d-DG of control mice was not altered by the application of SCH23390 (*p* > 0.05, *n* = 8) or L-sulpiride (*p* > 0.05, *n* = 8).

### MPTP Reduces the D1R-Mediated Survival of Newborn Neurons

**Figure 4:**
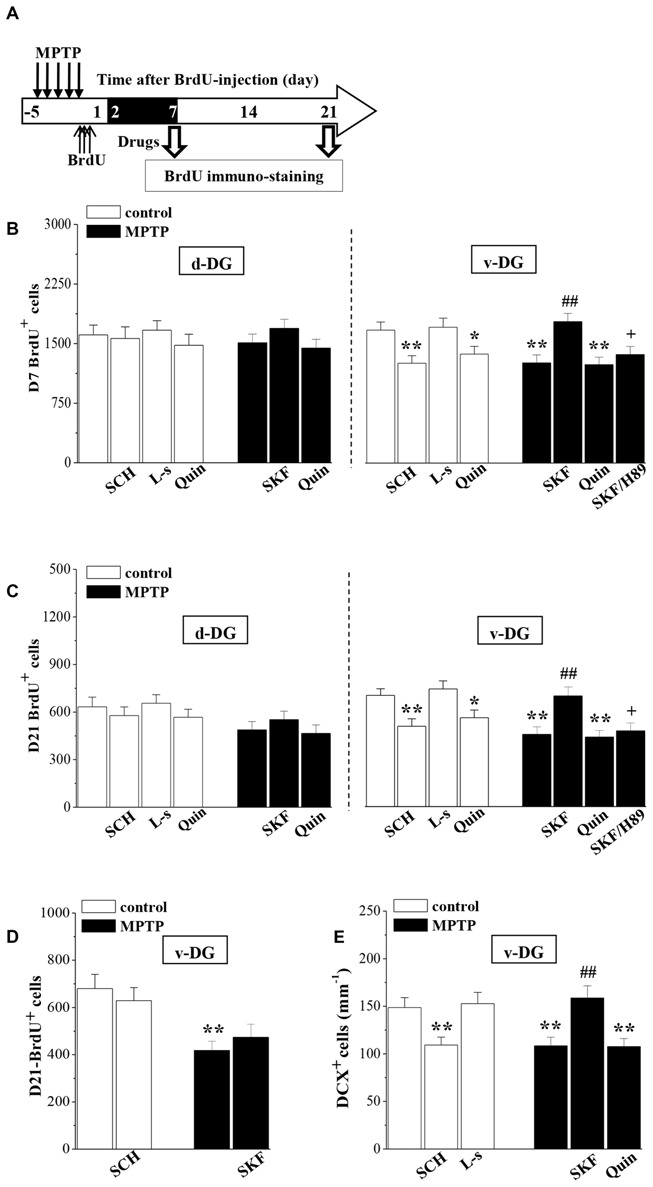
**Involvement of D1R and D2R in MPTP-impaired survival of newborn neurons. (A)** Time chart of the experimental procedure. The drugs were administered on days 2–7 after the BrdU-injection (black part). **(B,C)** Bar graph shows the mean number of D7-BrdU^+^ cells and D21-BrdU^+^ cells in d-DG and v-DG of MPTP-mice treated with SKF38393 (SKF), H89 or quinpirole (Quin) or control mice treated with SCH23390 (SCH), L-sulpiride (L-s) or Quin. *p < 0.05 and **p < 0.01 vs. control mice; ^##^*p* < 0.01 vs. MPTP-mice; ^ +^*p* < 0.05 vs. MPTP-mice treated with SKF. **(D)** Bar graph shows the mean number of D21-BrdU^+^ cells in v-DG when the drugs were administered on days 16–21 after the BrdU-injection. **p < 0.01 vs. control mice. **(E)** Bar graph shows the mean number of DCX^+^ cells in v-DG when the drugs were administered on days 2–7 post-MPTP. **p < 0.01 vs. control mice; ^##^*p* < 0.01 vs. MPTP-mice.

The newborn cells in the adult hippocampal DG express functional D1R and D2R (Takamura et al., 2014). To test whether the down-regulation of D1R-activated PKA-CREB signaling affects the survival of young newborn neurons, MPTP-mice were treated with SKF38393 or quinpirole daily on days 2–7 after the BrdU-injection, and control mice received the treatment with SCH23390, L-sulpiride or quinpirole. Then, we examined the number of D7- and D21-BrdU^+^ cells, respectively (Figure 4A). As shown in Figures 4B,C, the treatment with SKF38393 in MPTP-mice not only rescued the loss of D7-BrdU^+^ cells (*p* < 0.01, *n* = 10), but also corrected the reduction of D21-BrdU^+^ cells (*p* < 0.01, *n* = 10) in v-DG, which was sensitive to H89 (*p* < 0.05, *n* = 10). However, the administration of quinpirole to MPTP-mice had no effects on the reduced D7- or D21-BrdU^+^ cells in v-DG (*p* > 0.05, *n* = 10). Interestingly, the treatment of control mice with either SCH23390 (*p* < 0.01, *n* = 10) or quinpirole (*p* < 0.05, *n* = 10), but not L-sulpiride (*p* > 0.05, *n* = 10), caused a decrease in the number of D7-BrdU^+^ cells in v-DG, which was associated with the reduction of D21-BrdU^+^ cells (SCH23390: *p* < 0.01, *n* = 10; quinpirole: *p* < 0.05, *n* = 10). In contrast, the number of D7- or D21-BrdU^+^ cells in d-DG of control mice was not affected by SCH23390, quinpirole or L-sulpiride (*p* > 0.05, *n* = 10). However, the application of SKF38393 on days 16–21 after the BrdU-injection had no effect on the reduced D21-BrdU^+^ cells in v-DG of MPTP-mice (*p* > 0.05, *n* = 10; Figure 4D). At the same time, the treatment of control mice with SCH23390 did not change the number of D21-BrdU^+^ cells in v-DG (*p* > 0.05, *n* = 10). Additionally, the administration of SKF38393 for 6 days post-MPTP could increase the number of DCX^+^ cells in v-DG (*p* < 0.01, *n* = 10; Figure 4E), while the activation of D2R by quinpirole could not (*p* > 0.05, *n* = 10). Similarly, the treatment of control mice with SCH23390 (*p* < 0.01, *n* = 10), but not L-sulpiride (*p* > 0.05, *n* = 10), caused a reduction in the number of DCX^+^ cells in v-DG.

### Association of MPTP-Impaired Neurogenesis With Depressive-Like Behaviors

**Figure 5:**
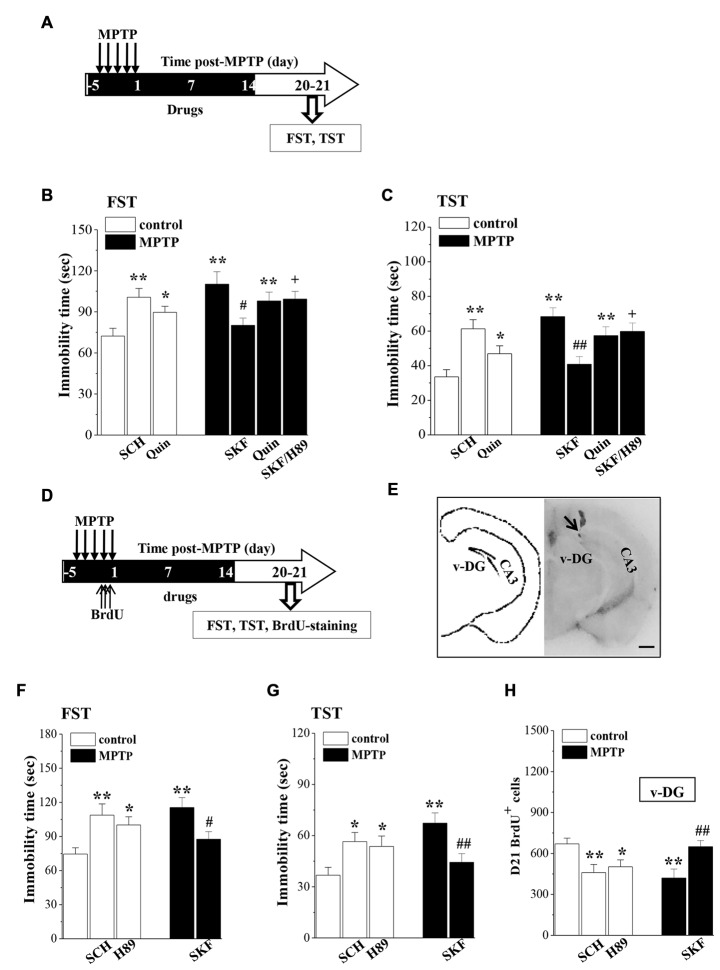
**Influence of MPTP-induced DA depletion on depressive-like behaviors. (A)** Time chart of SKF38393 (SKF), quinpirole (Quin), SCH23390 (SCH) and H89 injection (i.p.) for 18 days (black part). **(B,C)** Bar graphs show the immobility time in FST and TST on days 6–7 after the last-administration of drugs. *p < 0.05 and **p < 0.01 vs. control mice. ^#^*p* < 0.05 and ^##^*p* < 0.01 vs. MPTP-mice.^ +^*p* < 0.05 vs. MPTP-mice treated with SKF. **(D)** Time chart of v-DG injection of SKF38393 (SKF), SCH23390 (SCH) and H89 for 18 days (black part). **(E)** The representative Evans-blue image of v-DG injection (right panel) and schematic diagram of v-DG (left panel). Scale bar = 1.0 mm. Black arrow indicates the site of the v-DG injection (black spot). CA3: hippocampal CA3 region. **(F,G)** Bar graphs show the immobility time in FST and TST on days 6–7 after the v-DG last-injection of drugs. *p < 0.05 and **p < 0.01 vs. control mice. ^#^*p* < 0.05 and ^##^*p* < 0.01 vs. MPTP-mice. **(H)** Bar graph shows the mean number of D21-BrdU^+^ cells in v-DG. *p < 0.05 and **p < 0.01 vs. control mice. ^##^*p* < 0.01 vs. MPTP-mice.

To explore whether the impaired neurogenesis in MPTP-mice is related to their depressive-like behaviors, MPTP-mice were treated with SKF38393 or quinpirole for 18 days starting from the first-injection of MPTP, and control mice received SCH23390 and quinpirole for 18 days (Figure 5A). To avoid the direct effects of drugs, the affective behavioral tests were performed on days 6–7 after the end of the drugs administration. The results showed that the treatment of MPTP-mice with SKF38393, but not quinpirole, corrected the prolongation of the immobility time in FST (*p* < 0.05, *n* = 10; Figure 5B) and TST (*p* < 0.01, *n* = 10; Figure 5C), which was blocked by H89 (FST: *p* < 0.05; TST: *p* < 0.05, *n* = 10). In addition, the administration of SCH23390 (FST: *p* < 0.01; TST: *p* < 0.01, *n* = 10) or quinpirole (FST: *p* < 0.05; TST: *p* < 0.05, *n* = 10) to control mice caused an increase in the immobility time of FST and TST.

To determine further the relationship between the MPTP-impaired neurogenesis and the depressive-like behaviors, MPTP-mice were treated with the v-DG injection of SKF38393 (Figure 5E), and control mice received the v-DG injection of SCH23390 and H89 for 18 days (Figure 5D). Similarly, the v-DG injection of SKF38393 in MPTP-mice could prevent the prolongation of the immobility time in FST (*p* < 0.05, *n* = 10; Figure 5F) and TST (*p* < 0.01, *n* = 10; Figure 5G) and the reduction of D21-BrdU^+^ cells in v-DG (*p* < 0.01, *n* = 10; Figure 5H). The v-DG injection of SCH23390 and H89 in control mice caused the prolongation of the immobility time in FST (SCH23390: *p* < 0.01, *n* = 10; H89: *p* < 0.05, *n* = 10) and TST (SCH23390: *p* < 0.05, *n* = 10; H89: *p* < 0.05, *n* = 10) and the loss of D21-BrdU^+^ cells in v-DG (SCH23390: *p* < 0.01, *n* = 10; H89: *p* < 0.05, *n* = 10).

## Discussion

The present study provides evidence that the MPTP-induced decline of dopaminergic afferents in v-DG impairs the D1R-mediated early survival of newly generated neurons, and this deficit in hippocampal neurogenesis is associated with depressive-like behaviors.

The adult DG contains at least two types of proliferating immature cells: GFAP^+^ radial glia-like cell (B-cell) and GFAP^-^ cell (C-cells). The B-cells, as stem cells, generate frequently dividing transit-amplifying C-cells. Asymmetrically divided C-cells are more likely to be progenitor cells and can differentiate into neural precursors or DCX^+^ neuroblasts (A-cells; Zhao et al., 2008). D2R is predominantly expressed in C-cells, while A-cells express both D1R and D2R (Höglinger et al., 2004). The activation of D2R has been reported to stimulate C-cell proliferation (Yang et al., 2008). The number of D1-BrdU^+^ cells in v-DG or d-DG of MPTP-mice was not altered, although the dopaminergic neurons in VTA were reduced by approximately 25% on day 1 post-MPTP. The microtubule-associated protein DCX expression is specific to newly generated neurons, but not to glial cells and undifferentiated cells, which reaches a peak during the second week and is down-regulated concomitantly with the appearance of NeuN (Rao and Shetty, 2004). DCX, as an immature neuron marker, is used to evaluate the survival of young newborn neurons (Kim et al., 2009). MPTP-mice had a significant decrease in the DCX^+^ cells in v-DG, which could be rescued by the activation of D1R rather than D2R. In addition, the blockade of D1R caused a decrease of the DCX^+^ cells in v-DG of control mice. The number of D7-BrdU^+^ cells in v-DG of MPTP-mice was reduced. Although fewer D14- or D21-BrdU^+^ cells were found in v-DG of MPTP-mice than those in control mice, the percentage of reduced BrdU^+^ cells (D7 → −25% D14 → −57% D21) in MPTP-mice did not differ from control mice (D7 → −20% D14; D14→ −52% D21). Therefore, it is likely that the MPTP-induced DA depletion mainly impairs the early survival of newborn neurons in v-DG. Khaindrava et al. (2011) have reported that DA depletion causes deficits in the survival of newborn cells in DG. Our pharmacological experiments support this idea by showing that in MPTP-mice, the D1R agonist when administered on days 2–7 after BrdU-injection rescued the loss of D7- or D21-BrdU^+^ cells, but when administered on days 16–21 after BrdU-injection, it failed to alter the reduced D21-BrdU^+^ cells in MPTP-mice. Moreover, the blockade of D1R on days 2–7 after BrdU-injection reduced the number of D7- and D21-BrdU^+^ cells in control mice. The absolute number of D21-BrdU^+^/NeuN^+^ cells and proportion of these cells relative to the total D21-BrdU^+^ cells were reduced in v-DG of MPTP-mice. The number of BrdU^+^/GFAP^+^ cells in v-DG of MPTP-mice did not differ from control mice, and the ratio of BrdU^+^/GFAP^+^ cells was higher than the ratio in control mice. Thus, it is conceivable that the MPTP-induced DA depletion does not affect the differentiation of precursor cells. On the other hand, the number of BrdU^+^ cells, DCX^+^ cells, BrdU^+^/NeuN^+^ cells or BrdU^+^/GFAP^+^ cells in d-DG did not differ between control and MPTP-mice, which was not affected by the activation or blockade of D1R and D2R. Consistently with the report by Bjarkam et al. (2003), there was few dopaminergic projections in d-DG, indicating that the neurogenesis process in d-DG may be insensitive to the dopaminergic regulation.

The administration of D1R agonist to MPTP-mice could rescue the loss of D7-BrdU^+^ cells in a PKA-dependent manner, but the D2R agonist did not. The major signaling cascaded by D1R activation is the cAMP-PKA pathway in the hippocampus (Arnsten and Dudley, 2005). PKA-CREB signaling is one of the most important pathways for promoting neuron regeneration in DG (Miyamoto et al., 2009). The activation of PKA induces the phosphorylation of CREB at Ser-133, thereby facilitating the gene transcription of key molecules such as *c-Fos*, Jun-B, Bcl-2, GDNF and neurotrophins to regulate neuronal survival and regeneration. Inhibition of phosphodiesterase-4, an enzyme that catalyzes the hydrolysis of cAMP, stimulates the activation of CREB and increases the survival of newborn cells (Nakagawa et al., 2002). In this study, we observed the decline of PKA and CREB phosphorylation in v-DG of MPTP-mice without changes in the expression of D1R. In v-DG of MPTP-mice, the decrease of PKA and CREB phosphorylation could be corrected by the D1R agonist and the protection of D1R agonist on the D7-BrdU^+^ cells was blocked by the inhibition of PKA. The blockade of D1R caused the loss of D7-BrdU^+^ cells and decline of PKA and CREB phosphorylation in v-DG of control mice. The D2R antagonist haloperidol has been shown to decrease (Wakade et al., 2002), increase (Kippin et al., 2005) or not affect (Malberg et al., 2000) hippocampal neurogenesis. Our results showed that the activation of D2R reduced the PKA and CREB phosphorylation and the D7-BrdU^+^ cells in v-DG of control mice. Therefore, it is proposed that the MPTP-induced DA depletion through the down-regulation of D1R-induced PKA-CREB signaling impairs the early survival of newborn neurons in v-DG.

It is widely accepted that the hippocampal neurogenesis is required for mood control (Petrik et al., 2012) and cognitive performance (Rola et al., 2004). Selectively impairing the adult neurogenesis by telomerase inhibitor could cause the depression-like behaviors in mice (Zhou et al., 2011a). Irradiation of young animals impairs hippocampal neurogenesis that is associated with cognitive deficits (Rola et al., 2004). The newly generated neurons are integrated into the neuronal circuitry within 3–4 weeks after birth (Zhao et al., 2006) to regulate hippocampal output (Ming and Song, 2011). Importantly, we have observed the depressive-like behaviors in MPTP-mice for 2–3 weeks later after DA depletion. The timing of depressive-like behaviors in MPTP-mice seems to be coincident with the loss of mature newborn neurons. Interestingly, MPTP-mice did not appear affected by the spatial cognitive deficits, although they showed depressive-like behaviors. One possible explanation may be a difference in the regions of impaired neurogenesis in hippocampal DG. The idea may be supported by the facts that the lesions in the dorsal hippocampus affect spatial learning and memory (Moser et al., 1995), while lesions of the ventral hippocampus lead to anxiety and depressive-like behaviors (McHugh et al., 2004). The antidepressants may exert their behavioral effects by increasing neurogenesis in v-DG (Banasr et al., 2006). In particular, the v-DG injection of SKF38393 in MPTP-mice not only reduced the loss of D21-BrdU^+^ cells but also relieved the depressive-like behaviors. In control mice, the v-DG injection of either SCH23390 or H89 caused the reduction of D21-BrdU^+^ cells in v-DG, which was companied by the production of depressive-like behaviors. These results indicate a possible cause-and-effect relationship between the MPTP-impaired neurogenesis and the MPTP-induced affective disorder. In addition, the ventral hippocampus is involved in regulation of the hypothalamic-pituitary-adrenal axis (Herman et al., 1995), buffering the stress response (Snyder et al., 2011). The basolateral amygdaloid complex receives the hippocampal afferents (Supcun et al., 2012). Hippocampal dysfunction affects the synaptic plasticity of the basolateral amygdaloid complex, which is important for the acquisition and consolidation of fear memories (Goosens and Maren, 2002) and the extinction of learned fear (Dalton et al., 2012).

Forebrain DA circuitry has been studied by two largely independent researchers: a nigrostriatal DA system that originates in the substantia nigra (SN) and a mesolimbic DA system that originate in the VTA. MPTP selectively destroys the dopaminergic neurons in the SN and VTA (Lu et al., 2014; Hong et al., 2015; the present study). Target neurons in the primary terminal field of the SN are striatal medium spiny neurons. It is well known that the MPTP-induced loss of dopaminergic neurons in SN pars compacta through the striatal DA depletion impairs the motor functions including slowness, rigidity, resting tremor and postural instability (Fahn, 2003). The projection fields of VTA DA neurons are the hippocampus or medial prefrontal cortex, which contributes to the emotional regulation (Russo and Nestler, 2013). Tye et al. (2013) have reported that the selective inhibition of VTA DA neurons acutely induces multiple distinct depression-like behaviors. Furthermore, the dopaminergic fibers from VTA dopaminergic neurons have been reported to contact directly with the newborn cells in DG (Höglinger et al., 2004). Taken together, the results in the present study give an indication that the MPTP-impaired VTA dopaminergic neurons leading to the dopaminergic deficiency in v-DG, impairs the D1R-mediated early survival of newborn neurons.

The present study provides evidence that the MPTP-induced dopaminergic depletion impairs the D1R-mediated early survival of newly generated neurons in v-DG; this impairment of hippocampal neurogenesis is associated with the production of the depressive-like behaviors. Thus, D1R agonists may be candidate substrates for treating PD depression.

## Author Contributions

In this study, TZ performed the immuno-staining, western blotting and all statistical analysis. JH finished the behavioral examination and RT-PCR analysis. TD carried out the animal care. LC designed the experiment and finished the manuscript.

## Funding

This study was supported by the National 973 Basic Research Program of China (2014CB943303) and the National Natural Science Foundation of China (81471157; 81671253).

## Conflict of Interest Statement

The authors declare that the research was conducted in the absence of any commercial or financial relationships that could be construed as a potential conflict of interest.
